# Improving early infant HIV diagnosis in Kenya: study protocol of a cluster-randomized efficacy trial of the HITSystem

**DOI:** 10.1186/s13012-015-0284-3

**Published:** 2015-07-09

**Authors:** Sarah Finocchario-Kessler, Kathy Goggin, Samoel Khamadi, Brad Gautney, Jacinda K. Dariotis, Charles Bawcom, An-Lin Cheng, Niaman Nazir, Catherine Martin, Andrea Ruff, Michael Sweat, Vincent Okoth

**Affiliations:** Department of Family Medicine, University of Kansas Medical Center, Kansas City, KS USA; Children’s Mercy Hospitals and Clinics, Health Services and Outcomes Research, University of Missouri-Kansas City, Schools of Medicine and Pharmacy, Kansas City, MO USA; Kenya Medical Research Institute, Nairobi, Kenya; Walter Reed U.S. Military HIV Research Program, Mbeya, Tanzania; Global Health Innovations, Kansas City, MO USA; Johns Hopkins Bloomberg School of Public Health, Baltimore, MD USA; University of Cincinnati, Cincinnati, OH USA; School of Nursing, University of Missouri-Kansas City, Kansas City, MO USA; Medical University of South Carolina, Psychiatry and Behavioral Sciences, Charleston, SC USA

**Keywords:** Early infant diagnosis, HIV-exposed infants, Kenya, HITSystem

## Abstract

**Background:**

Early infant diagnosis among human immunodeficiency virus (HIV)-exposed infants is a critical component of prevention of mother-to-child transmission programs. Barriers to early infant diagnosis include poor uptake, low retention at designated re-testing intervals, delayed test results, passive systems of communication, and poor linkage to treatment. This study will evaluate the HIV Infant Tracking System (HITSystem), an eHealth intervention that streamlines communication and accountability between the key early infant diagnosis stakeholders: HIV+ mothers and their HIV-exposed infants, healthcare providers, and central laboratory personnel. It is hypothesized that the HITSystem will significantly improve early infant diagnosis retention at 9 and 18 months postnatal and the timely provision of services.

**Methods/design:**

Using a phased cluster-randomized controlled trial design, we will evaluate the impact of the HITSystem on eight primary benchmarks in the 18-month long cascade of care for early infant diagnosis. Study sites are six government hospitals in Kenya matched on geographic region, resource level, and patient volume. Early infant diagnosis outcomes of mother-infant dyads (*n* = 120 per site) at intervention hospitals (*n* = 3) where the HITSystem is deployed at baseline will be compared to the matched control sites providing standard care. After allowing for sufficient time for enrollment and 18-month follow-up of dyads, the HITSystem will be deployed at the control sites in the end of Year 3. Primary outcomes are retention among mother-infant dyads, initiation of antiretroviral therapy among HIV-infected infants, and the proportion of services delivered within the optimal time window indicated by national and study guidelines. Satisfaction interviews with participants and providers will inform intervention improvements. Cost-effectiveness analyses will be conducted to inform the sustainability of the HITSystem. Hypothesized outcomes include significantly higher retention throughout the 18-month early infant diagnosis process, significantly more services provided on-time at intervention sites, and a potential savings to the healthcare system.

**Discussion:**

This study will evaluate the public health impact of the HITSystem to improve critical early infant diagnosis outcomes in low-resource settings. Cost-effectiveness analyses will inform the feasibility of scale-up in other settings.

**Trial registration:**

ClinicalTrials.gov: NCT02072603

## Background

While early infant diagnosis (EID) and prompt treatment of human immunodeficiency virus (HIV)+ infants are critical components of prevention of mother-to-child transmission (PMTCT) efforts, EID has received minimal attention compared to other HIV prevention and treatment services. The primary objective of EID is to promptly identify HIV-infected infants to initiate live-saving antiretroviral therapy (ART) as soon as possible [1, 2]. Beyond facilitating prompt ART initiation, EID provides critical reassurance to families whose infants are uninfected and allows implementation of feeding plans designed to minimize risk of transmission or optimize survival among those identified as HIV-infected [3]. Similarly, EID and infant feeding methods inform postnatal ART prophylaxis recommendations [4].

EID care and management includes an algorithm and series of interventions known as the “EID cascade of care”. This cascade includes the following: (1) the offer and acceptance of EID testing among HIV-exposed infants, (2) accurate specimen collection, transport, and laboratory processing; (3) relay of results to both healthcare providers and mothers/caregivers; and (4) linkage to care: OI prophylaxis for all HIV-exposed infants (cotrimoxazole for *pneumocystis jiroveci* pneumonia) and ART for HIV+ infants [5]. Because HIV infection cannot be definitively excluded until at least 6 weeks after cessation of breastfeeding, re-testing infants who initially test negative is critical to identify instances of postnatal HIV transmission. Kenyan EID guidelines call for routine re-testing of HIV-exposed infants at 9 and 18 months postnatal [6].

Although the guidelines provide clear objectives, challenges at every step in the EID cascade of care compromise the quality of EID services provided in Kenya and other low-resource settings [5, 7–9]. Infant testing often occurs after the recommended 6 weeks of age (average age of 3.1 months) [8, 10, 11], and turn-around times for results are typically quite long (average of 1–3 months) [10]. Retention throughout the recommended 18-month postnatal period is low (32–74.7 %) [8, 12] and less than one-third of Kenya’s HIV-infected infants are initiated on ART [6, 13]. Transportation, documentation, and communication challenges contribute to lost or delayed samples [5, 7], and a passive system for mother notification or follow-up leads to delayed care and missed opportunities to support retention [14–16]. Limited coordination between EID stakeholders limits accountability, efficiency, and linkage to treatment [5, 17]. In addition to these system-level challenges, mothers experience individual barriers that limit their full participation in the EID process (e.g., lack of: awareness of the existence or importance of EID services, resources for transportation, and fear of disclosure of their HIV status) [18, 19].

Our study aims to evaluate the HIV Infant Tracking System (HITSystem), a web-based tool designed to address many of the barriers in current EID service provision in Kenya, with the ultimate goal of maximizing the quality of EID services. As a system-level intervention, it was not deemed feasible or acceptable to randomize at the individual level; therefore, randomization occurred at the site (hospital) level. Primary outcomes will be measured at the participant (mother-infant dyad) level and aggregated to report outcomes per hospital. The primary outcomes for efficacy of the HITSystem are retention through the EID Cascade of Care (measured by eight unique benchmarks between 0–18 months postnatal, including ART initiation among HIV-infected infants) (Table 1) and efficiency of services (turn-around times for EID services and ability to meet age and time-specific targets with the HITSystem compared to control sites where standard of care services are provided). Secondary outcomes are maternal and infant predictors of retention, postnatal period(s) at the highest risk for loss to follow-up, satisfaction among mothers and providers, and infant HIV infection status over time. Cost-effectiveness analyses will quantify resources required to implement the HITSystem versus standard EID services and calculate potential short and long-term costs averted by this intervention to inform scale-up and sustainability.

**Table 1:** HITSystem targets: eight time-sensitive EID interventions

1.Initiation of OI prophylaxis at 6 weeks
2.Collect dried blood spot (DBS) for PCR test by 6 weeks
3.Receipt of DBS at lab within 10 days of collection
4.Return of PCR results from lab within 2 weeks
5.Notify mother within 2 weeks of the EID provider receiving results
6.Initiate all HIV-infected infants on ART within 4 weeks of notifying the mother
7.Retest all HIV-uninfected infants at 9 months, initiate ART w/in 4 weeks if applicable^a^
8.Retest all HIV-uninfected infants at 18 months, initiate ART w/in 4 weeks if applicable^a^

^a^Re-testing indicated at these time points or 6 weeks after cessation of breastfeeding

Greater detail regarding the 8 intervention points and the HITSystem process has been previously published. [21]

## Methods/design

### Trial design

This is a 5-year phased cluster-randomized, controlled trial design to rigorously evaluate the impact of the HITSystem in Kenya. Study sites are six government hospitals in Kenya matched on geographic region, resource level, and patient volume to produce three-matched pairs. One hospital in each pair was randomly assigned to either the intervention (HITSystem) or control group (standard of care) condition; resulting in three interventions and three control study sites. We anticipate a 12-month window to complete enrollment of *n* = 120 mother-infant dyads per hospital, followed by an 18-month follow-up period (until end of Year 3). In Year 4 the HITSystem will cross over to initial control sites whose standard of care data from the 12 months prior will serve as a comparison for analyses.

### Recruitment, participants, and setting

This study is being conducted at government hospitals at the provincial, county, and sub-county levels in Kenya. The study protocol is in compliance with the Helsinki Declaration and was reviewed and approved by the Institutional Review Boards at the Kenya Medical Research Institute (protocol #2726) and the University of Kansas Medical Center (protocol #13793). Matched hospitals meet the following selection criteria: government funded; provide PMTCT, EID, and ART services; have at least one dedicated EID provider; and maintain medical files for HIV-exposed infants. Matched hospitals reflect regional differences in HIV prevalence, geography (e.g., average travel distance to clinic, accessible transportation), and cultural influences (e.g., tribal practices, religious beliefs).

### Participant eligibility and consent

As a system-level intervention, any HIV-infected mother whose infant is <18 months at the time of initial enrollment in EID and presents for care through the Maternal and Child Health (MCH) department is eligible to be enrolled in the HITSystem. Mothers <18 years of age or who present for care through other departments can utilize the HITSystem, but their data will be exempted from analyses. Cell phone ownership is not a requirement for participation. Mothers are informed about the HITSystem during enrollment by trained research or clinical staff, and those wanting to participate provide written informed consent prior to enrollment or completion of the baseline survey. A separate informed consent is conducted prior to the satisfaction interview near the time of 9-month re-testing. Those who wish to opt out receive the paper-based standard of care. Based on our pilot experience, we anticipate >95 % participation.

### Standard of care

The current standard of care (SOC) for EID in Kenya has many well-documented challenges [8, 10, 11, 17]; however, when effectively implemented as designed, the following should take place: The EID provider should gather information on relevant maternal and infant clinical and demographic data at the time of postnatal enrollment in EID. These data should be entered in a large paper registry book (HIV-Exposed Infant Registry), organized by month of birth. Dried blood spot samples should be collected at the targeted age of 6 weeks and sent via courier to the central laboratory for processing. The samples should then be processed at the lab and paper polymerase chain reaction (PCR) test results returned to the hospital via courier and then sent to MCH to be added to the mother-infant file and recorded in the EID registry. If the infant has an HIV-positive test result, EID staff should contact the mother via phone to ask her to return to the clinic where she should be counseled about the HIV-positive result and referred to the Comprehensive Care Center (CCC) where a first line pediatric ART regimen should be initiated [6, 20]. If the result of the initial PCR test is negative, the result should be noted in the paper registry and the mother should be informed at a subsequent clinic visit and advised to return with the infant for a re-test at 9 months of age. At 9-months postnatal, all HIV-exposed infants should receive an antibody test, and if positive, a confirmatory PCR test should be conducted using the previously described process for PCR testing. If the antibody result is negative, the mother should be asked to return 6 weeks after the cessation of breastfeeding or when the infant is 18 months old to repeat this process. Even when implemented as designed, the current SOC for EID has multiple gaps where mothers often fall out of care, employ extremely limited prospective tracking to identify when services are indicated, and rely on a passive system for communicating test results to mothers. These shortcomings combined with well-documented challenges involving delayed or lost samples resulting in long turn-around time for infant test results combine to produce the observed poor retention and completion rates [8, 10, 11, 17].

## Trial intervention

### HIV infant tracking system

The HITSystem was designed to overcome barriers in providing high quality EID in low-resource settings by utilizing available technology to facilitate timely communication and accountability between primary stakeholders in the EID process. The primary goals of the HITSystem are the following: to (a) improve EID utilization and clinical management of HIV-exposed infants, (b) facilitate early ART initiation for infants identified as HIV-infected, and c) improve efficiency in providing time-sensitive interventions. The HITSystem is accessed online through a computer, using mobile broadband connections. Reliance on cellular signals (readily available and reliable in nearly all areas) rather than hard wired internet access makes this system feasible even in remote areas. The primary components include the following: (1) tracking of infants to increase retention, (2) action alerts to complete time-sensitive interventions, (3) real time communication of PCR lab results to hospitals, and (4) real time communication with mothers regarding required follow-up care via text messaging or patient tracing.

The HITSystem tracks the care of infants enrolled in EID so that those who drop out of care can be easily identified and targeted for outreach. Using the infant’s date of birth, the HITSystem configures automated electronic alerts for eight time-sensitive interventions to optimize care for HIV-exposed infants. The order and timing of these action alerts have been previously published [21]. PCR test results entered into the online system from the lab become available to hospitals in “real time” thus eliminating the need for costly and unreliable couriers to transport paper results. Excessive delays for laboratory test results are also targeted by triggering alerts at the laboratory for samples received but not tested within 10 days. When results are available, mothers are sent an Short Message Service (SMS) message asking them to come to the hospital at their earliest convenience. Text messages do not relay sensitive information that could compromise confidentiality. In the case of indeterminate results, mothers are notified to return with infants to collect another sample. To address one of the weakest areas of current EID care, the HITSystem proactively texts mothers when re-testing of infants is due at 9 and 18 months of age. Infants enrolled in the HITSystem are tracked until they are either (a) conclusively determined HIV negative at 18 months and discharged from EID, (b) determined to be HIV-infected, initiated on ART, and linked to pediatric HIV care, or (c) dead or transfered to another health facility.

#### HITSystem staffing

At each participating hospital, the EID provider enters relevant data into the HITSystem during initial enrollment and subsequent visits. EID providers at each hospital monitor and respond to the action alerts for enrolled infants and coordinate participant tracing for those mothers without phones. This process is supported by a Research Coordinator at each intervention site. At laboratories, one to two technicians are trained on the HITSystem and serve as a liaison for follow-up requests made by EID staff or the Research Coordinator. The Study Manager reviews action alerts to identify systematic challenges across hospitals, provide quality assurance with data collection, and provide refresher training with hospital and lab-based staff every 6 months or earlier as needs arise due to program modifications or personnel changes. Once deployed, the HITSystem will continue in the setting until the end of the study. A phase-out plan will be implemented to maximize adoption among site staff and sustained use after the study period.

### Training at study sites

#### Control sites (standard of care)

There is no training or intervention regarding EID procedures at control sites. Nurses directly involved in EID enrollment were trained to assess participant eligibility, to conduct informed consent, and administer the brief baseline survey. The same nurses were trained on procedures to ensure participant confidentiality and secure storage of study data.

#### Intervention sites (HITSystem)

Training for providers began with a discussion of the challenges and barriers to EID care and an introduction to how the HITSystem is designed to tackle these issues; doing this was designed to promote buy-in from the facility staff and generate enthusiasm about the introduction of a new tool. The intuitive and user-friendly nature of the HITSystem allows training of key clinic/laboratory personnel in 1 to 2 days. During initial training, new users were educated about the HITSystem’s standard operating procedures, and hands-on data entry scenarios were used to tailor training of each staff member to the specific capacity in which s/he would utilize the HITSystem. Research team members received extensive HITSystem training to ensure their ability to respond effectively to any user-challenges and provide refresher training as needed. Furthermore, they receive ongoing training on study protocols, informed consent, and data collection and data management procedures to ensure rigorous conduct of the study protocol.

### Participant enrollment

When a mother first presents her infant for EID, the EID provider creates a new infant record in the EID Registry notebook (SOC) or the HITSystem (intervention), and data regarding her antenatal care, maternal ARV prophylaxis regimens, and postpartum infant prophylaxis are entered. The mother’s cell phone number is also recorded in the HITSystem at this time. The majority of mothers have cell phones (97 % in urban and 82 % in peri-urban pilot hospitals) (Finocchario-Kessler et al., Lessons learned from implementing the HIV Infant Tracking System (HITSystem) to improve early infant diagnosis of HIV outcomes in Kenya, submitted) [22], but for the few mothers without access or who prefer not to use their cell phones, we ask for a hand drawn map from the clinic to their home or major landmark to assist follow-up efforts. At subsequent consultations (PCR test results, 9 month re-testing, etc.), the infant’s information is updated in the EID Registry notebook (SOC) or pulled up electronically on the HITSystem and populated with additional data (intervention).

### Fidelity assurance procedures

Standard operating procedures were developed for each step of the study process to standardize training, enrollment, and all data collection procedures. Because it is web-based, the intervention features remain uniform across all sites utilizing the HITSystem. Furthermore, any modification or corrections to the HITSystem are instantly applied across all users. HITSystem entries are routinely reviewed to ensure completeness and appropriate utilization of the comments section. Site Coordinators make monthly visits to the control sites to cross reference enrollment records with the EID Registry and to review informed consent documents and surveys for completeness and secure storage. A random sub-sample of informed consents and surveys are reviewed by the Study Manager for accuracy and completeness at each site.

### Randomization

Prior to randomization, 12 hospitals were surveyed to determine the location, patient volume and HIV prevalence (through review of EID Registry for prior 12 months), consistency in current EID procedures, and feasibility of study procedures. Using these data, six sites with the most comparable characteristics and regional location were selected. All individuals enrolled in the study at each hospital comprised the unit or cluster for randomization. A random number generator program was then used to randomly assign control and intervention sites within a given geographical region. Study statistician was blind to the process of choosing the sites. Similarly, all research staff was blind to the randomization process.

### Study measures and data sources

This system-level intervention is implemented at the site level, but outcome measures pertain to both the participant and cluster level. Primary data collection efforts include the following: (1) retrospective data collection of EID outcomes prior to study implementation, (2) baseline surveys at the time of enrollment, (3) ongoing collection of EID outcome data during the study period, (4) satisfaction surveys with mothers and providers, and (5) collection of EID-related costs.

#### Retrospective data

Research Coordinators conducted a thorough retrospective record review of the EID Registry at each hospital to document EID outcomes (Table 1) during the 12 months prior to study implementation. Supporting medical records (described in the primary EID outcome section below) were used to complete missing data points in the EID Registry.

#### Baseline surveys

A brief, one-time baseline survey was conducted with each mother at the time of EID enrollment at intervention and control sites. Surveys collected basic demographic data and a variety of potential baseline predictors of EID retention, including the following: EID knowledge, stigma/violence, motivation and self-efficacy for completing EID, partner support, disclosure, distance and cost to the hospital, income, education, and number of children.

#### Primary EID outcomes

The HITSystem output provides infant date of birth, dates of services (sample collection, sample processed and result posted, notification of mother, treatment initiation or re-testing as applicable, discharge), and outcomes of service (infant test results at each time period, initiation of ART, reason for discharge). To assess the standard provision of EID care at control sites, all infants entered in the HIV-Exposed Infant Registry Book each month during the enrollment period are reviewed until the sample size of *n* = 120 mother-infant dyads is achieved per control site. Consistent with the strategy employed and deemed feasible in the pilot study [21], and used for the retrospective data collection, we cross reference the following sources: (1) infant medical records, (2) hospital HIV-Exposed Infant Registry, (3) Clinical Care Center (CCC) records, and (4) central laboratory PCR database to permit parallel assessment of the outcomes collected by the HITSystem. The following EID outcomes are measured at all sites.

##### EID retention

EID retention will be characterized as complete or incomplete. Incomplete EID care (loss to follow-up) is defined as one or more of the following: (1) DBS not collected, (2) mother not notified of result, (3) HIV-infected infant not started on ART, (4) HIV-uninfected infant not re-tested at 9 months, and 5) HIV-uninfected infant not re-tested at 18 months.

##### Time to ART initiation

In addition to calculating the proportion of HIV-infected infants who are initiated on ART, we will calculate the number of days between the date that the mother was notified of her infant’s HIV-positive status and the date the infant was initiated on ART.

##### Turn-around time of infant test results

We will calculate the number of weeks between the date that the dried blood spot sample was collected and the date that the result was made available to the hospital. We will also calculate the time (in weeks) to notifying the mother of her infant’s test results.

##### Timely delivery of EID Services

For each of the 8 EID intervention points (Table 1), we will create an Optimal EID Care Index to measure whether the service was completed at all (1 point) or never completed (0 point) and whether it was completed on-time (1 point) or off-time (0 point). For each EID intervention point, each mother-infant dyad may receive either 0 point (never completed), 1 point (off-time completion), or 2 points (on-time completion). From these data, we will create an Optimal EID Care Index score for each dyad by summing across the 8 EID intervention point scores (range of 0–16 points). Dyads receiving a minimum score of 0 will have completed none of the 8 EID intervention points. Dyads receiving a maximum score of 16 will have completed all 8 points on-time. Scores between 0 and 16 will reflect some combination of completion and on-time completion.

#### Participant satisfaction

A sub-sample of mothers at all intervention and control sites (*n* = 30 per site) will complete satisfaction interviews when their infants are between 9 and 18 months of age. EID providers at each site and laboratory technicians at participating laboratories will also complete satisfaction interviews after 6–12 months of study implementation. Purposive sampling includes mothers with both HIV-infected and uninfected infants and those with and without mobile phones. Semi-structured surveys will elicit mothers’ experiences with the HITSystem and standard EID care. Satisfaction among EID providers and laboratory technicians will be assessed to identify perceived strengths and weaknesses, motivations and confidence to provide EID services, and recommendations to improve future EID outcomes.

#### Cost-effectiveness

We will evaluate the cost-effectiveness of the HITSystem from a “donor” or government perspective to evaluate integration of the HITSystem technology into an existing EID system. Cost data will be collected three times over the course of the study: 6, 18, and 30 months after intervention launch. A costing survey documents facility and laboratory related costs for commodities and services rendered and estimates low, medium, and high ranges for each type of expense. Relevant costing data from mothers is collected by the enrollment survey and again by the satisfaction surveys.

### Reimbursement

As a system-level intervention, all HIV-infected mothers and their HIV-exposed infants (mother-infant pairs) are eligible to participate. No remuneration is provided for enrollment in the HITSystem or completion of enrollment and satisfaction surveys. Satisfaction surveys are coordinated to coincide with scheduled appointments to avoid any additional transport burdens. Record review for cost-effectiveness analyses is conducted by study staff and thus do not require remuneration.

### Statistical analyses

We will calculate descriptive statistics to compare outcomes for each of the eight time-sensitive EID outcomes by intervention or control hospitals. In our preliminary analyses, we will estimate general linear models to determine whether there are pre-intervention differences across conditions and hospitals—for any of the measures we will be using as predictors or outcomes. Any significantly different variables will be included as covariates in our analytic models. Given attrition (not completing all 8 EID intervention points) is a major outcome of interest, we will predict and describe it. Hence, even “missing” or “lost” dyads will be included in outcome measures.

To predict patterns of EID utilization among HIV-exposed infants, we will first identify bivariate correlates of the main outcome: EID retention. Correlations between incomplete EID retention and possible predictors (e.g., infant sex, maternal disclosure, etc.) will be conducted and tested for significance using Phi, Point Biserial, or Spearman Rho statistics depending on the measurement level of each predictor. We will then include variables that are significantly correlated with EID retention in the generalized linear mixed model and use a stepwise approach to identify a parsimonious list of predictors, controlling for the correlation of observations within cluster with random effects. We will follow the same steps to assess correlates of the 5 unique EID intervention points that comprise the summative incomplete EID retention variable:(1) DBS not collected, (2) mother not notified of result, (3) HIV+ infant not started on ART, (4) HIV infant not re-tested at 9 months, and (5) HIV infant not re-tested at 18 months. To identify the most vulnerable time periods for EID retention, we will use survival analysis with random effects [23] to determine how long infants stay in EID care and identify time periods when significant drops in EID retention occur. Visual EID retention curves will illustrate differences in EID care retention at various time points throughout the 18-month EID period. Comparisons between intervention and control participants will highlight the impact of the HITSystem at various time points in EID utilization.

To assess the impact of the HITSystem on timely administration of eight critical EID interventions (Optimal EID Care Index), we will analyze data using multilevel modeling (Proc MIXED in the SAS System version 9.4) [24] to account for the nesting of time within dyad within hospital. This will increase the precision of treatment effect estimates by more correctly estimating standard errors (that are not correctly estimated when nesting is ignored) and indicate inferences about change over time by condition. We will include covariates that may be associated with EID completion (e.g., infant sex, distance from hospital, cell phone ownership) in addition to any pre-intervention differences identified across conditions and hospitals. Outcome variables of interest include an aggregate score and sub-scale scores (based on the Optimal EID Care Index), infant age at first PCR test, proportion re-tested by 9 months, and proportion re-tested by 18 months.

#### Power and sample size

To be conservative, the sample size calculation for the analyses to determine intervention efficacy for retention and ART initiation is based on the smallest effect size obtained in our pilot work, 0.44 (range 0.44–1.98). Using Murray’s method to conduct power analysis for group-randomized trials, we accounted for the number of groups, participants in each group, and intraclass correction (ICC) estimated at 0.05 [25]. This calculation required *n* = 120 mother-infants dyads from each of the six participant hospitals for a total of 720 infants (360 from intervention hospitals and 360 from control hospitals). A sample of 720 infants is sufficient to detect with 80 % power, an effect size of 0.44 with an ICC range from 0.01 to 0.05. Due to multiple planned comparisons, a more conservative alpha level of 0.01 will adjust for possible inflated type I error. Given the EID volume and low refusal rates anticipated (<1 % in the pilot study), the required sample size for each hospital should be met within 12 months of enrollment.

#### Qualitative analyses

Qualitative satisfaction data will be translated and transcribed prior to content analyses to identify emergent themes from each stakeholder group. Data will be analyzed utilizing a web-based program called Dedoose, and themes will be discussed and confirmed by study staff members to reach consensus. We will use standard procedures for estimation of the cost of intervention [26] that adhere to the Panel on Cost-Effectiveness in Health and Medicine’s guidelines, prior to removing the cost saved from the intervention (e.g., cost of medical care that would have been spent on HIV-infected infants who were not directly linked to ART due to the intervention). We will also calculate the laboratory costs saved by avoiding unnecessary PCR tests as a result of improved intervention communication, record keeping, and patient retention.

### Trial status

We are approaching the end of study Year 2 and are actively enrolling participants and collecting data. No analyses have been conducted at this time.

## Discussion

While previous efforts have been made to improve EID through the utilization of various interventions targeting only one of the key players in the EID system (most often the hospital or laboratory) [11, 16, 27–30], the HITSystem is the first system to comprehensively link multiple stakeholders (health facilities, mother-infant pairs, and laboratories) with an interactive tool that facilitates multidirectional communication, data management, and outcomes monitoring.

The web-based nature of the system minimizes the impact of geographical challenges to the current system, and this intervention relies heavily on the current communications infrastructures, reducing the need for costly hardware and system upgrades. This study will allow an analysis of outcomes at each step of the EID cascade of care and will facilitate identification of specific challenges within the participating facilities. Previous studies related to system-level interventions addressing EID have been limited to observational or quasi-experimental studies in which historical data served as the basis for comparison. The rigor of this study, as a RCT, greatly increases the value of the comparative data and will facilitate identification of improvements related to the intervention versus those resulting from the maturation of EID practices over time.

The intervention is applicable to all HIV-exposed infants and their mothers/guardians, given its adaptability regardless of cell phone ownership or literacy. However, it is only able to address outcomes in those mothers who present themselves for care at facilities. The intervention targets quality of care for clinic-based populations, and the study makes no attempt to directly target EID uptake. Although the study focuses on a system-level intervention, the HITSystem’s ability to capture and analyze individual factors that may influence EID retention will form the basis for exploration in further studies. Given the rapid pace of technological innovation in Kenya, the clean study design may be challenged by the introduction of other eHealth interventions at study sites that may influence existing processes for care. Every effort will be made to control for external influences in the design and analyses.

## Abbreviations

ART: antiretroviral therapy
CCC: Comprehensive Care Clinic
EID: early infant diagnosis
HAART: highly active antiretroviral therapy
HITSystem: HIV infant tracking system
HIV: human immunodeficiency virus
ICC: intraclass correction
MCH: Maternal and Child Health
PCR: polymerase chain reaction
PMTCT: prevention of mother-to-child transmission of HIV
SAS: Statistical Application Software
SOC: standard of care
SOP: standard operating procedures
SMS: Short Message Service
